# Supplementary material for: Maternal-fetal bonding among pregnant women at psychosocial risk: The roles of adult attachment style, prenatal parental reflective functioning, and depressive symptoms
Source: PLoS One. 2020 Sep 17;15(9):e0239208. doi: 10.1371/journal.pone.0239208 (PMC7498041; doi:10.1371/journal.pone.0239208)
Supplement: S1 File — (PDF) [file pone.0239208.s001.pdf]

Avoidant attachment and anxious attachment and MAAS quality
Test for linearity assumption

Dependent Variable: MAAS_Q_Blm

Source	DF	Type III SS	Mean Square	F Value	Pr > F
ECRS_Av_BLm	1	35.56127478	35.56127478	2.45	0.1220
ECRS_Av_B*ECRS_Av_BL	1	29.63202135	29.63202135	2.04	0.1574
ECRS_A*ECRS_A*ECRS_A	1	26.79311980	26.79311980	1.84	0.1786
ECRS*ECRS*ECRS*ECRS_	1	25.42271929	25.42271929	1.75	0.1899

Avoidant attachment and anxious attachment and MAAS quality
Test for linearity assumption

Dependent Variable: MAAS_Q_Blm

Source	DF	Type III SS	Mean Square	F Value	Pr > F
ECRS_Av_BLm	1	16.30211574	16.30211574	1.11	0.2952
ECRS_Av_B*ECRS_Av_BL	1	7.04918423	7.04918423	0.48	0.4904
ECRS_A*ECRS_A*ECRS_A	1	2.95479679	2.95479679	0.20	0.6549

Avoidant attachment and anxious attachment and MAAS quality
Test for linearity assumption

Dependent Variable: MAAS_Q_Blm

Source	DF	Type III SS	Mean Square	F Value	Pr > F
ECRS_Av_BLm	1	90.18890749	90.18890749	6.21	0.0149
ECRS_Av_B*ECRS_Av_BL	1	59.39014709	59.39014709	4.09	0.0467

Avoidant attachment and anxious attachment and MAAS quality
Association between variable and outcome

Number of Observations Read	78
Number of Observations Used	78

Dependent Variable: MAAS_Q_Blm

Source	DF	Sum of Squares	Mean Square	F Value	Pr > F
Model	1	101.310284	101.310284	6.71	0.0115
Error	76	1147.966861	15.104827		
Corrected Total	77	1249.277145			

R-Square	Coeff Var	Root MSE	MAAS_Q_Blm Mean
0.081095	8.822370	3.886493	44.05271

Source	DF	Type III SS	Mean Square	F Value	Pr > F
ECRS_Av_BLm	1	101.3102837	101.3102837	6.71	0.0115

Parameter	Estimate	Standard Error	t Value	Pr > |t|	95% Confidence Limits	
Intercept	46.72712589	1.12252142	41.63	<.0001	44.49143074	48.96282105
ECRS_Av_BLm	-0.23977553	0.09258401	-2.59	0.0115	-0.42417256	-0.05537850

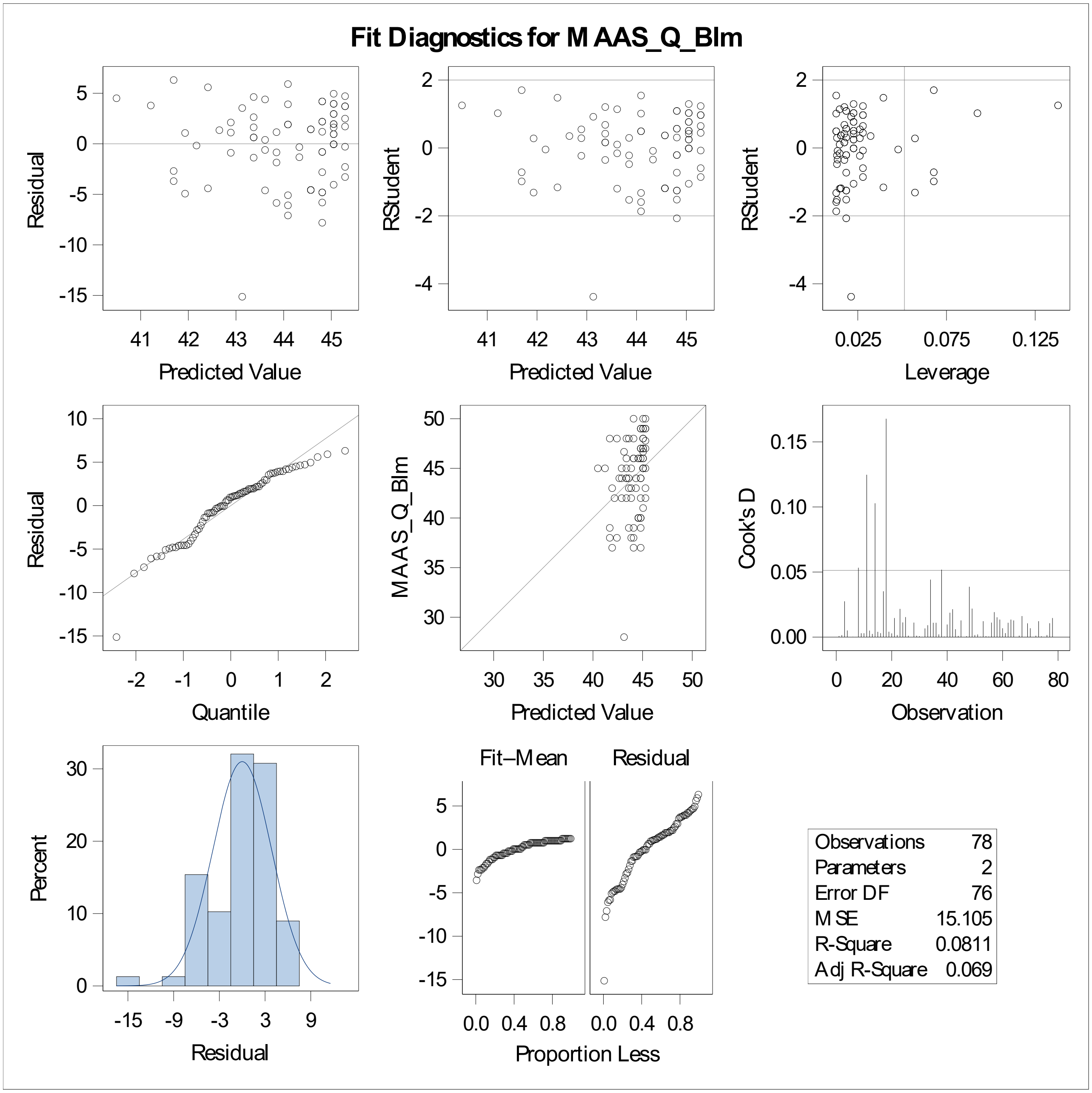

Avoidant attachment and anxious attachment and MAAS quality
Test for linearity assumption

Dependent Variable: MAAS_Q_Blm

Source	DF	Type III SS	Mean Square	F Value	Pr > F
ECRS_An_BLm	1	12.27166374	12.27166374	0.77	0.3821
ECRS_An_B*ECRS_An_BL	1	8.85771431	8.85771431	0.56	0.4574
ECRS_A*ECRS_A*ECRS_A	1	5.17189114	5.17189114	0.33	0.5699
ECRS*ECRS*ECRS*ECRS_	1	2.41907992	2.41907992	0.15	0.6974

Avoidant attachment and anxious attachment and MAAS quality
Test for linearity assumption

Dependent Variable: MAAS_Q_Blm

Source	DF	Type III SS	Mean Square	F Value	Pr > F
ECRS_An_BLm	1	36.39664827	36.39664827	2.32	0.1320
ECRS_An_B*ECRS_An_BL	1	47.72845654	47.72845654	3.04	0.0853
ECRS_A*ECRS_A*ECRS_A	1	56.86470003	56.86470003	3.62	0.0608

Avoidant attachment and anxious attachment and MAAS quality
Test for linearity assumption

Dependent Variable: MAAS_Q_Blm

Source	DF	Type III SS	Mean Square	F Value	Pr > F
ECRS_An_BLm	1	31.23803586	31.23803586	1.92	0.1696
ECRS_An_B*ECRS_An_BL	1	28.88273471	28.88273471	1.78	0.1863

Avoidant attachment and anxious attachment and MAAS quality
Association between variable and outcome

Number of Observations Read	78
Number of Observations Used	78

Dependent Variable: MAAS_Q_Blm

Source	DF	Sum of Squares	Mean Square	F Value	Pr > F
Model	1	2.525877	2.525877	0.15	0.6959
Error	76	1246.751268	16.404622		
Corrected Total	77	1249.277145			

R-Square	Coeff Var	Root MSE	MAAS_Q_Blm Mean
0.002022	9.194127	4.050262	44.05271

Source	DF	Type III SS	Mean Square	F Value	Pr > F
ECRS_An_BLm	1	2.52587650	2.52587650	0.15	0.6959

Parameter	Estimate	Standard Error	t Value	Pr > |t|	95% Confidence Limits	
Intercept	44.61737977	1.51035259	29.54	<.0001	41.60925189	47.62550765
ECRS_An_BLm	-0.02874968	0.07326728	-0.39	0.6959	-0.17467412	0.11717476

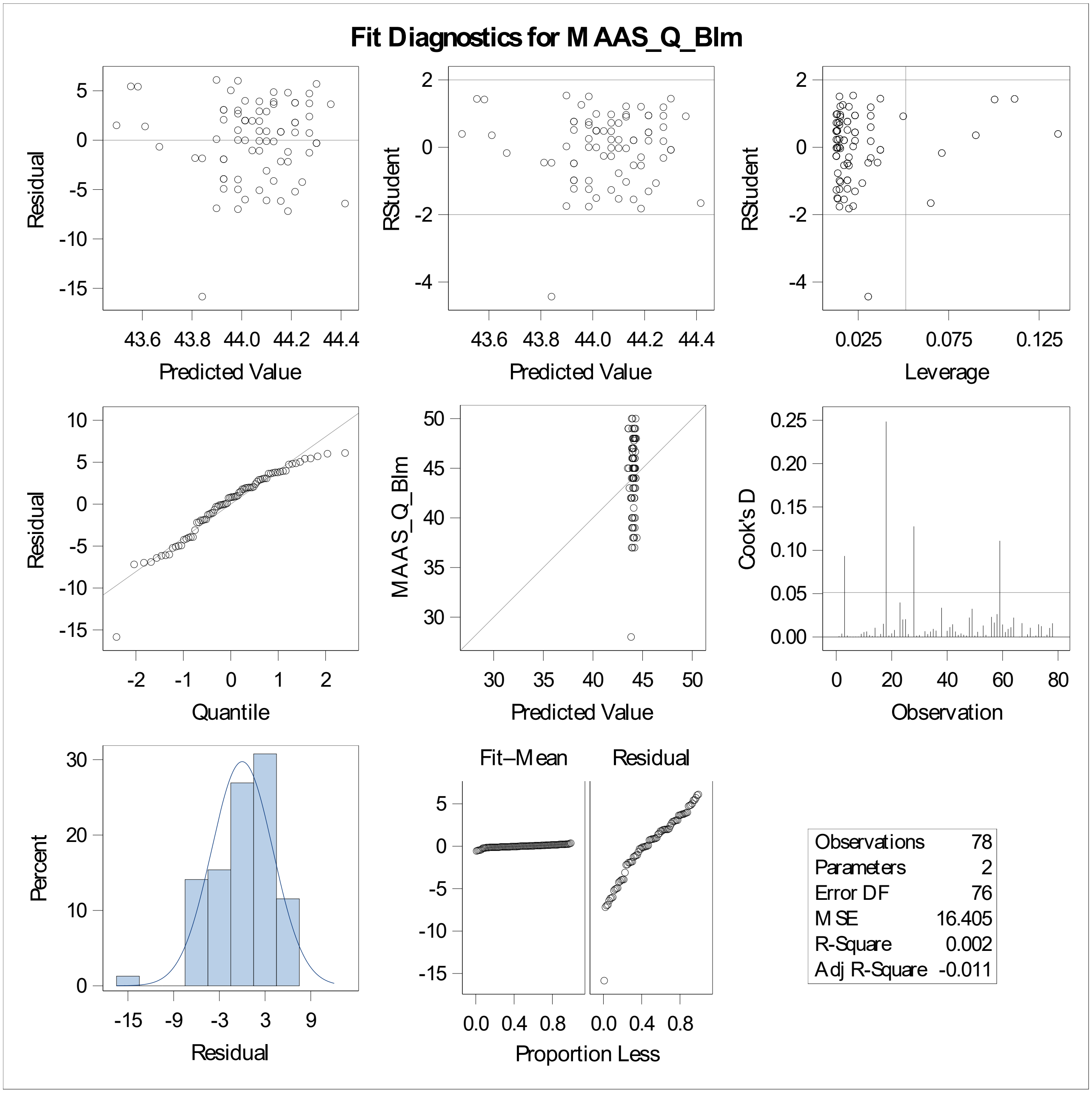

Avoidant attachment and anxious attachment and MAAS quality
Test for linearity assumption

Dependent Variable: MAAS_Q_Blm

Source	DF	Type III SS	Mean Square	F Value	Pr > F
EPDS_BLm	1	33.28257425	33.28257425	2.37	0.1280
EPDS_BLm*EPDS_BLm	1	26.49202933	26.49202933	1.89	0.1737
EPDS_B*EPDS_B*EPDS_B	1	30.27849917	30.27849917	2.16	0.1462
EPDS*EPDS*EPDS*EPDS_	1	34.31651344	34.31651344	2.44	0.1223

Avoidant attachment and anxious attachment and MAAS quality
Test for linearity assumption

Dependent Variable: MAAS_Q_Blm

Source	DF	Type III SS	Mean Square	F Value	Pr > F
EPDS_BLm	1	2.01022183	2.01022183	0.14	0.7089
EPDS_BLm*EPDS_BLm	1	2.40053413	2.40053413	0.17	0.6833
EPDS_B*EPDS_B*EPDS_B	1	5.27919732	5.27919732	0.37	0.5455

Avoidant attachment and anxious attachment and MAAS quality
Test for linearity assumption

Dependent Variable: MAAS_Q_Blm

Source	DF	Type III SS	Mean Square	F Value	Pr > F
EPDS_BLm	1	54.55017778	54.55017778	3.84	0.0537
EPDS_BLm*EPDS_BLm	1	10.59340542	10.59340542	0.75	0.3904

Avoidant attachment and anxious attachment and MAAS quality
Association between variable and outcome

Number of Observations Read	78
Number of Observations Used	78

Dependent Variable: MAAS_Q_Blm

Source	DF	Sum of Squares	Mean Square	F Value	Pr > F
Model	1	174.235241	174.235241	12.32	0.0008
Error	76	1075.041904	14.145288		
Corrected Total	77	1249.277145			

R-Square	Coeff Var	Root MSE	MAAS_Q_Blm Mean
0.139469	8.537551	3.761022	44.05271

Source	DF	Type III SS	Mean Square	F Value	Pr > F
EPDS_BLm	1	174.2352409	174.2352409	12.32	0.0008

Parameter	Estimate	Standard Error	t Value	Pr > |t|	95% Confidence Limits	
Intercept	46.54201385	0.82729980	56.26	<.0001	44.89430351	48.18972420
EPDS_BLm	-0.29285968	0.08344446	-3.51	0.0008	-0.45905372	-0.12666565

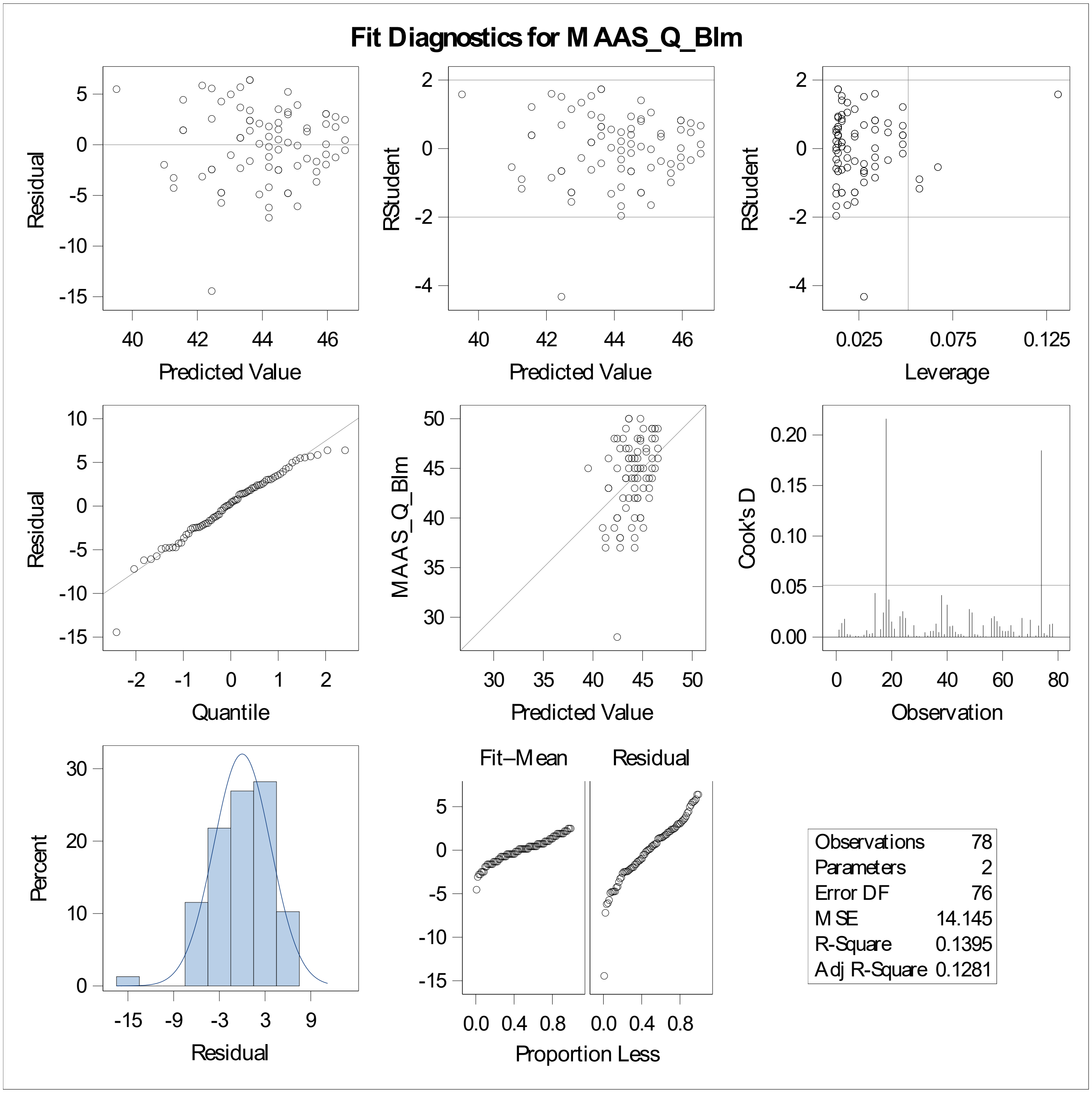

Avoidant attachment and anxious attachment and MAAS quality
Test for linearity assumption

Dependent Variable: MAAS_Q_Blm

Source	DF	Type III SS	Mean Square	F Value	Pr > F
PPRFQ_BLm	1	11.28266225	11.28266225	0.74	0.3929
PPRFQ_BLm*PPRFQ_BLm	1	10.28658313	10.28658313	0.67	0.4146
PPRFQ_*PPRFQ_*PPRFQ_	1	9.25161250	9.25161250	0.61	0.4390
PPRF*PPRF*PPRF*PPRFQ	1	8.42705064	8.42705064	0.55	0.4600

Avoidant attachment and anxious attachment and MAAS quality
Test for linearity assumption

Dependent Variable: MAAS_Q_Blm

Source	DF	Type III SS	Mean Square	F Value	Pr > F
PPRFQ_BLm	1	7.27053087	7.27053087	0.48	0.4911
PPRFQ_BLm*PPRFQ_BLm	1	7.41864628	7.41864628	0.49	0.4867
PPRFQ_*PPRFQ_*PPRFQ_	1	6.32167770	6.32167770	0.42	0.5208

Avoidant attachment and anxious attachment and MAAS quality
Test for linearity assumption

Dependent Variable: MAAS_Q_Blm

Source	DF	Type III SS	Mean Square	F Value	Pr > F
PPRFQ_BLm	1	1.46344362	1.46344362	0.10	0.7562
PPRFQ_BLm*PPRFQ_BLm	1	6.23962381	6.23962381	0.41	0.5218

Avoidant attachment and anxious attachment and MAAS quality
Association between variable and outcome

Number of Observations Read	78
Number of Observations Used	78

Dependent Variable: MAAS_Q_Blm

Source	DF	Sum of Squares	Mean Square	F Value	Pr > F
Model	1	113.121262	113.121262	7.57	0.0074
Error	76	1136.155883	14.949420		
Corrected Total	77	1249.277145			

R-Square	Coeff Var	Root MSE	MAAS_Q_Blm Mean
0.090549	8.776868	3.866448	44.05271

Source	DF	Type III SS	Mean Square	F Value	Pr > F
PPRFQ_BLm	1	113.1212616	113.1212616	7.57	0.0074

Parameter	Estimate	Standard Error	t Value	Pr > |t|	95% Confidence Limits	
Intercept	36.39197147	2.81910603	12.91	<.0001	30.77723521	42.00670773
PPRFQ_BLm	0.11750980	0.04271832	2.75	0.0074	0.03242890	0.20259070

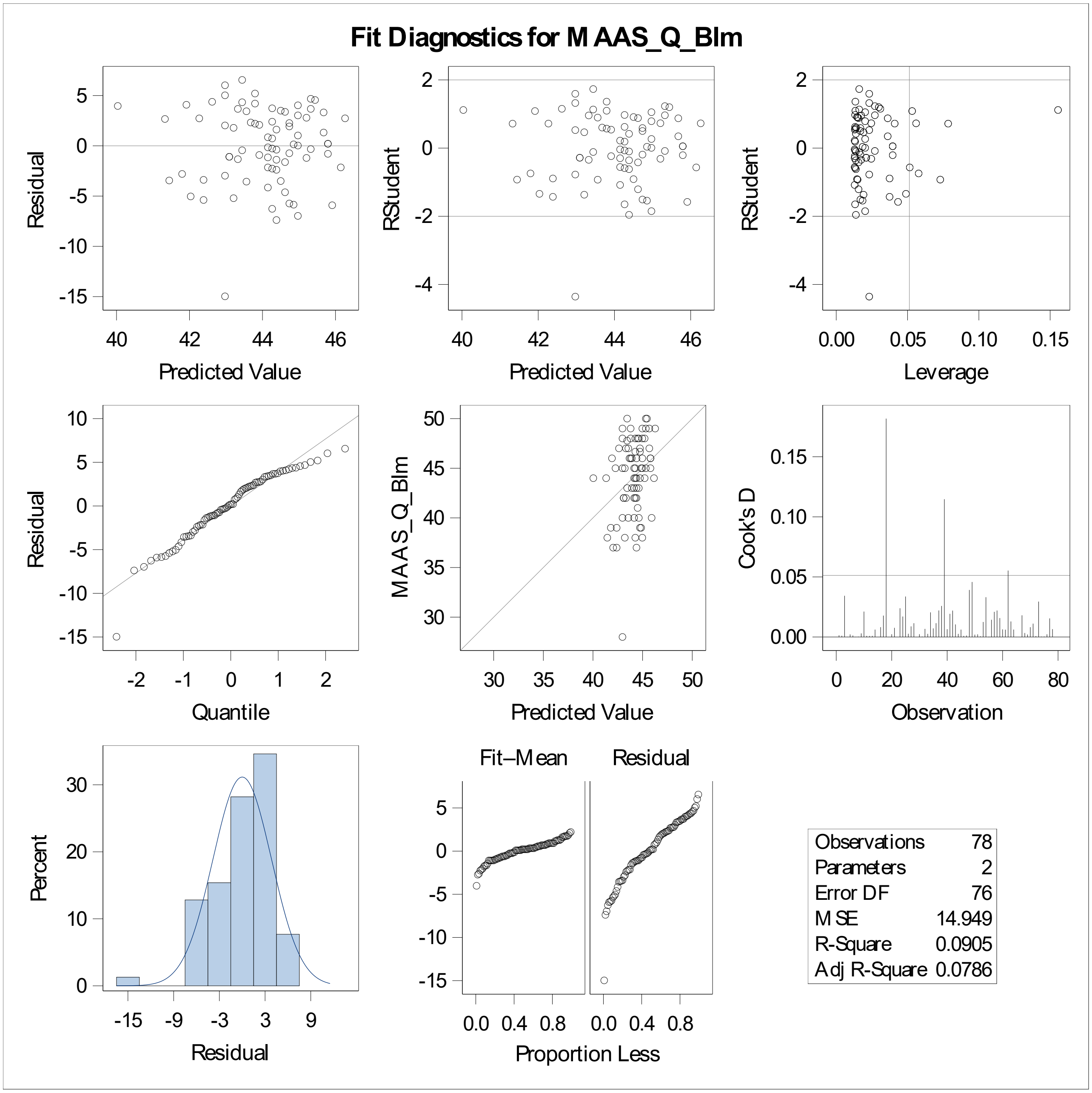

Avoidant attachment and anxious attachment and MAAS quality
Test for linearity assumption

Dependent Variable: MAAS_Q_Blm

Source	DF	Type III SS	Mean Square	F Value	Pr > F
GA	1	3.45253894	3.45253894	0.22	0.6438
GA*GA	1	1.73220654	1.73220654	0.11	0.7431
GA*GA*GA	1	0.71494508	0.71494508	0.04	0.8332
GA*GA*GA*GA	1	0.20178194	0.20178194	0.01	0.9109

Avoidant attachment and anxious attachment and MAAS quality
Test for linearity assumption

Dependent Variable: MAAS_Q_Blm

Source	DF	Type III SS	Mean Square	F Value	Pr > F
GA	1	52.27428662	52.27428662	3.31	0.0729
GA*GA	1	45.86429900	45.86429900	2.90	0.0926
GA*GA*GA	1	40.45354254	40.45354254	2.56	0.1138

Avoidant attachment and anxious attachment and MAAS quality
Test for linearity assumption

Dependent Variable: MAAS_Q_Blm

Source	DF	Type III SS	Mean Square	F Value	Pr > F
GA	1	35.51261416	35.51261416	2.20	0.1420
GA*GA	1	31.31326024	31.31326024	1.94	0.1676

Avoidant attachment and anxious attachment and MAAS quality
Association between variable and outcome

Number of Observations Read	78
Number of Observations Used	78

Dependent Variable: MAAS_Q_Blm

Source	DF	Sum of Squares	Mean Square	F Value	Pr > F
**Model**	1	8.629838	8.629838	0.53	0.4694
**Error**	76	1240.647307	16.324307		
**Corrected Total**	77	1249.277145			

R-Square	Coeff Var	Root MSE	MAAS_Q_Blm Mean
0.006908	9.171593	4.040335	44.05271

Source	DF	Type III SS	Mean Square	F Value	Pr > F
**GA**	1	8.62983787	8.62983787	0.53	0.4694

Parameter	Estimate	Standard Error	t Value	Pr > |t|	95% Confidence Limits	
**Intercept**	42.68983149	1.92946114	22.13	<.0001	38.84697658	46.53268640
**GA**	0.08545358	0.11752935	0.73	0.4694	-0.14862640	0.31953356

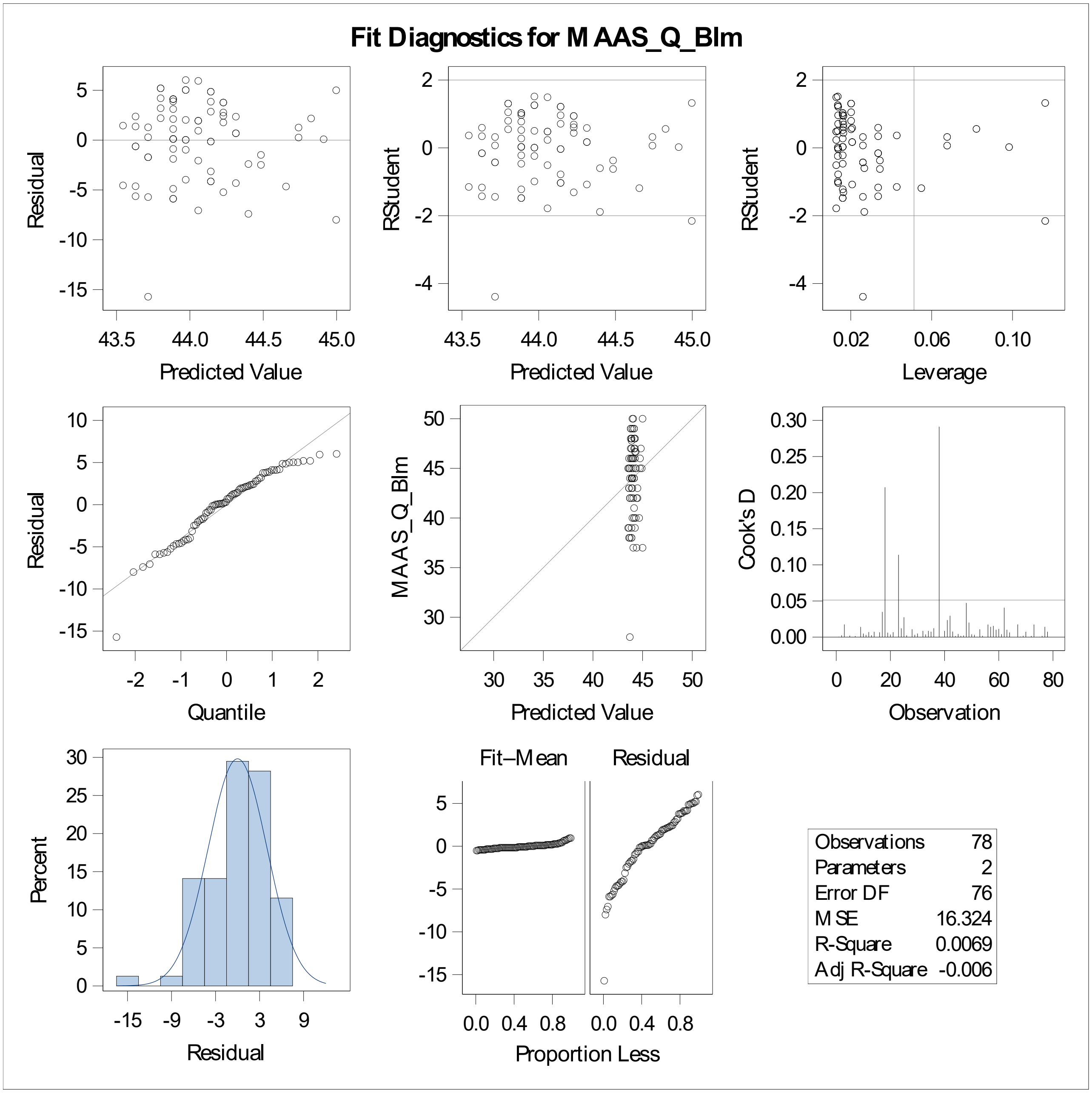

Avoidant attachment and anxious attachment and MAAS quality
Test for linearity assumption

Dependent Variable: MAAS_Q_Blm

Source	DF	Type III SS	Mean Square	F Value	Pr > F
**Mage**	1	30.23807024	30.23807024	2.04	0.1574
**Mage*Mage**	1	26.99417853	26.99417853	1.82	0.1813
**Mage*Mage*Mage**	1	23.14714550	23.14714550	1.56	0.2154
**Mage*Mage*Mage*Mage**	1	19.13419339	19.13419339	1.29	0.2596

Avoidant attachment and anxious attachment and MAAS quality
Test for linearity assumption

Dependent Variable: MAAS_Q_Blm

Source	DF	Type III SS	Mean Square	F Value	Pr > F
Mage	1	56.47729644	56.47729644	3.80	0.0552
Mage*Mage	1	66.79182605	66.79182605	4.49	0.0375
Mage*Mage*Mage	1	75.39462212	75.39462212	5.07	0.0273

Avoidant attachment and anxious attachment and MAAS quality
Test for linearity assumption

Dependent Variable: MAAS_Q_Blm

Source	DF	Type III SS	Mean Square	F Value	Pr > F
Mage	1	51.71471939	51.71471939	3.30	0.0734
Mage*Mage	1	43.57691781	43.57691781	2.78	0.0997

Avoidant attachment and anxious attachment and MAAS quality
Association between variable and outcome

Number of Observations Read	78
Number of Observations Used	78

Dependent Variable: MAAS_Q_Blm

Source	DF	Sum of Squares	Mean Square	F Value	Pr > F
Model	1	29.335982	29.335982	1.83	0.1804
Error	76	1219.941163	16.051857		
Corrected Total	77	1249.277145			

R-Square	Coeff Var	Root MSE	MAAS_Q_Blm Mean
0.023482	9.094735	4.006477	44.05271

Source	DF	Type III SS	Mean Square	F Value	Pr > F
Mage	1	29.33598154	29.33598154	1.83	0.1804

Parameter	Estimate	Standard Error	t Value	Pr > |t|	95% Confidence Limits	
Intercept	47.57496977	2.64465621	17.99	<.0001	42.30768043	52.84225910
Mage	-0.11406391	0.08437438	-1.35	0.1804	-0.28211006	0.05398223

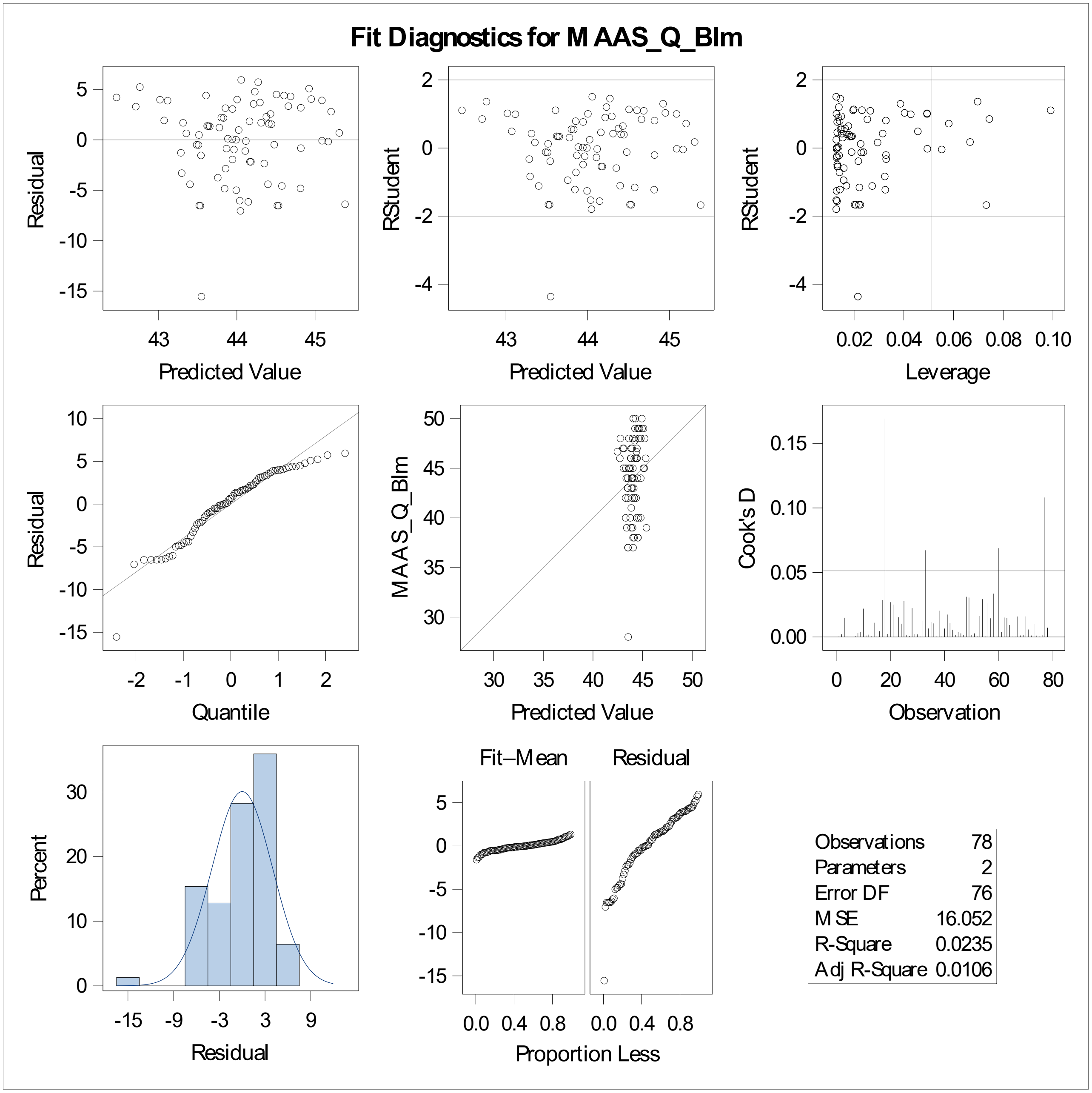

Model fit plots

Results model 1

Class Level Information		
**Class**	**Levels**	**Values**
**Edu2way**	2	0 1
**Par**	2	0 1

**Number of Observations Read**	78
**Number of Observations Used**	78

Dependent Variable: MAAS_Q_Blm

Source	DF	Sum of Squares	Mean Square	F Value	Pr > F
**Model**	6	185.285968	30.880995	2.06	0.0687
**Error**	71	1063.991177	14.985791		
**Corrected Total**	77	1249.277145			

R-Square	Coeff Var	Root MSE	MAAS_Q_Blm Mean
0.148315	8.787539	3.871149	44.05271

Source	DF	Type III SS	Mean Square	F Value	Pr > F
**ECRS_Av_BLm**	1	112.4912249	112.4912249	7.51	0.0078
**ECRS_An_BLm**	1	0.0240212	0.0240212	0.00	0.9682
**Edu2way**	1	41.9831452	41.9831452	2.80	0.0986
**Par**	1	1.6798581	1.6798581	0.11	0.7388
**Mage**	1	3.8410287	3.8410287	0.26	0.6142
**GA**	1	11.4829996	11.4829996	0.77	0.3843

Parameter	Estimate		Standard Error	t Value	Pr > |t|	95% Confidence Limits	
**Intercept**	45.72880782	B	3.88583014	11.77	<.0001	37.98068259	53.47693306
**ECRS_Av_BLm**	-0.26106357		0.09528550	-2.74	0.0078	-0.45105745	-0.07106969
**ECRS_An_BLm**	0.00287657		0.07184846	0.04	0.9682	-0.14038520	0.14613834
**Edu2way 0**	1.57341417	B	0.94003800	1.67	0.0986	-0.30096837	3.44779671
**Edu2way 1**	0.00000000	B	.	.	.	.	.

Parameter		Estimate		Standard Error	t Value	Pr > |t|	95% Confidence Limits	
**Par**	**0**	0.33307073	B	0.99480972	0.33	0.7388	-1.65052350	2.31666497
**Par**	**1**	0.00000000	B	.	.	.	.	.
**Mage**		-0.04617621		0.09120829	-0.51	0.6142	-0.22804037	0.13568796
**GA**		0.09924767		0.11337896	0.88	0.3843	-0.12682356	0.32531890

Note: The X'X matrix has been found to be singular, and a generalized inverse was used to solve the normal equations. Terms whose estimates are followed by the letter 'B' are not uniquely estimable.

Results model 2

Class Level Information		
**Class**	**Levels**	**Values**
**Edu2way**	2	0 1
**Par**	2	0 1

**Number of Observations Read**	78
**Number of Observations Used**	78

Dependent Variable: MAAS_Q_Blm

Source	DF	Sum of Squares	Mean Square	F Value	Pr > F
**Model**	8	402.187596	50.273449	4.10	0.0005
**Error**	69	847.089549	12.276660		
**Corrected Total**	77	1249.277145			

R-Square	Coeff Var	Root MSE	MAAS_Q_Blm Mean
0.321936	7.953669	3.503807	44.05271

Source	DF	Type III SS	Mean Square	F Value	Pr > F
**ECRS_Av_BLm**	1	31.2376037	31.2376037	2.54	0.1153
**ECRS_An_BLm**	1	0.9124749	0.9124749	0.07	0.7860
**Edu2way**	1	17.6339246	17.6339246	1.44	0.2348
**Par**	1	5.8317285	5.8317285	0.48	0.4930
**Mage**	1	0.9507850	0.9507850	0.08	0.7816
**GA**	1	14.2647046	14.2647046	1.16	0.2848

Source	DF	Type III SS	Mean Square	F Value	Pr > F
EPDS_BLm	1	97.4011836	97.4011836	7.93	0.0063
PPRFQ_BLm	1	127.0410600	127.0410600	10.35	0.0020

Parameter		Estimate		Standard Error	t Value	Pr > |t|	95% Confidence Limits	
Intercept		37.44729083	B	4.41967774	8.47	<.0001	28.63027498	46.26430669
ECRS_Av_BLm		-0.15206573		0.09533066	-1.60	0.1153	-0.34224520	0.03811374
ECRS_An_BLm		-0.01926647		0.07066947	-0.27	0.7860	-0.16024821	0.12171527
Edu2way	0	1.04140029	B	0.86892706	1.20	0.2348	-0.69206176	2.77486233
Edu2way	1	0.00000000	B	.	.	.	.	.
Par	0	-0.65809743	B	0.95484232	-0.69	0.4930	-2.56295573	1.24676088
Par	1	0.00000000	B	.	.	.	.	.
Mage		-0.02328289		0.08366346	-0.28	0.7816	-0.19018693	0.14362115
GA		0.11111799		0.10308444	1.08	0.2848	-0.09452983	0.31676581
EPDS_BLm		-0.25919256		0.09201964	-2.82	0.0063	-0.44276672	-0.07561840
PPRFQ_BLm		0.14748722		0.04584822	3.22	0.0020	0.05602253	0.23895192

Note: The X'X matrix has been found to be singular, and a generalized inverse was used to solve the normal equations. Terms whose estimates are followed by the letter 'B' are not uniquely estimable.

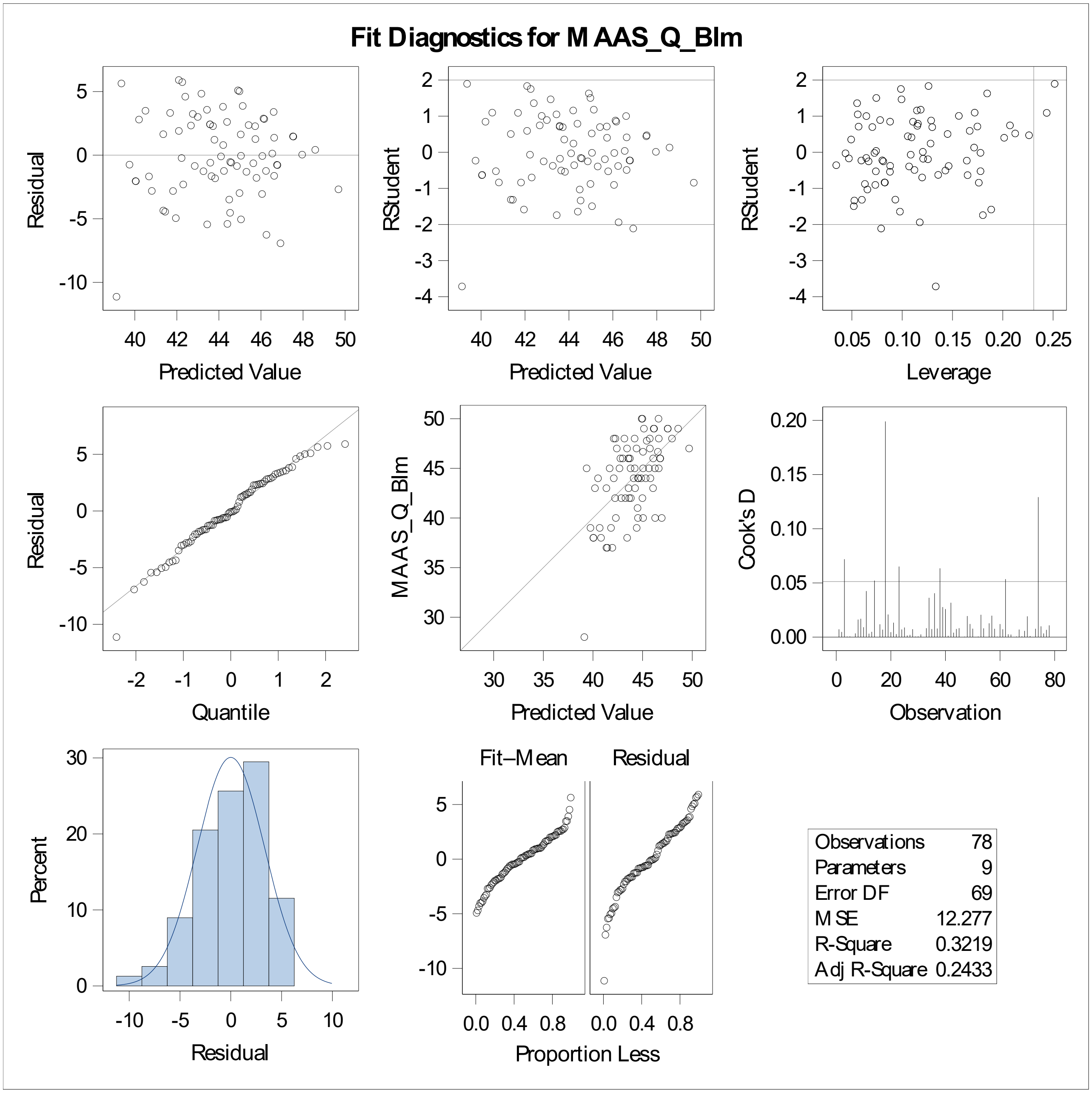

Standardized variables

Variable	N	Mean	Std Dev
MAAS_Q_Blm_Stand	78	-1.174132E-8	1.0000000
MAAS_P_Blm_Stand	78	2.4011668E-9	1.0000000
ECRS_An_Blm_stand	78	6.5121999E-9	1.0000000
ECRS_av_BLm_stand	78	-9.647868E-9	1.0000000
EPDS_Blm_stand	78	-6.40513E-18	1.0000000
PPRFQ_Blm_stand	78	-7.45768E-10	1.0000000
Edu2way_stand	78	-7.665552E-8	1.0000000
Par_stand	78	-5.101939E-8	1.0000001
Mage_stand	78	-8.80817E-9	1.0000000
GA_stand	78	1.2435501E-8	1.0000000

Standardized variables
Model 1

Number of Observations Read	78
Number of Observations Used	78

Dependent Variable: MAAS_Q_Blm_Stand

Source	DF	Sum of Squares	Mean Square	F Value	Pr > F
Model	6	11.42021955	1.90336993	2.06	0.0687
Error	71	65.57977910	0.92365886		
Corrected Total	77	76.99999865			

R-Square	Coeff Var	Root MSE	MAAS_Q_Blm_Stand Mean
0.148315	-8.18538E9	0.961072	-0.000000

Source	DF	Type III SS	Mean Square	F Value	Pr > F
ECRS_An_Blm_stand	1	0.00148056	0.00148056	0.00	0.9682
ECRS_av_BLm_stand	1	6.93346885	6.93346885	7.51	0.0078
Edu2way_stand	1	2.58765810	2.58765810	2.80	0.0986
Par_stand	1	0.10353913	0.10353913	0.11	0.7388
Mage_stand	1	0.23674427	0.23674427	0.26	0.6142
GA_stand	1	0.70776205	0.70776205	0.77	0.3843

Parameter	Estimate	Standard Error	t Value	Pr > |t|	95% Confidence Limits	
Intercept	-.0000000337	0.10881994	-0.00	1.0000	-.2169808398	0.2169807724
ECRS_An_Blm_stand	0.0044990297	0.11237278	0.04	0.9682	-.2195659252	0.2285639846
ECRS_av_BLm_stand	-.3100550337	0.11316687	-2.74	0.0078	-.5357033627	-.0844067047
Edu2way_stand	-.1959937604	0.11709668	-1.67	0.0986	-.4294779180	0.0374903972
Par_stand	-.0415577991	0.12412409	-0.33	0.7388	-.2890542088	0.2059386106
Mage_stand	-.0620355847	0.12253409	-0.51	0.6142	-.3063616274	0.1822904581
GA_stand	0.0965299250	0.11027425	0.88	0.3843	-.1233506942	0.3164105441

Standardized variables

Model 2

**Number of Observations Read**	78
**Number of Observations Used**	78

Dependent Variable: MAAS_Q_Blm_Stand

Source	DF	Sum of Squares	Mean Square	F Value	Pr > F
**Model**	8	24.78909060	3.09863632	4.10	0.0005
**Error**	69	52.21090805	0.75667983		
**Corrected Total**	77	76.99999865			

R-Square	Coeff Var	Root MSE	MAAS_Q_Blm_Stand Mean
0.321936	-7.40865E9	0.869873	-0.000000

Source	DF	Type III SS	Mean Square	F Value	Pr > F
**ECRS_An_Blm_stand**	1	0.05624098	0.05624098	0.07	0.7860
**ECRS_av_BLm_stand**	1	1.92534975	1.92534975	2.54	0.1153
**Edu2way_stand**	1	1.08687826	1.08687826	1.44	0.2348
**Par_stand**	1	0.35944233	0.35944233	0.48	0.4930
**Mage_stand**	1	0.05860224	0.05860224	0.08	0.7816
**GA_stand**	1	0.87921423	0.87921423	1.16	0.2848
**EPDS_Blm_stand**	1	6.00338447	6.00338447	7.93	0.0063
**PPRFQ_Blm_stand**	1	7.83025727	7.83025727	10.35	0.0020

Parameter	Estimate	Standard Error	t Value	Pr > |t|	95% Confidence Limits	
**Intercept**	-.0000000204	0.09849377	-0.00	1.0000	-.1964897223	0.1964896815
**ECRS_An_Blm_stand**	-.0301332394	0.11052880	-0.27	0.7860	-.2506321719	0.1903656932
**ECRS_av_BLm_stand**	-.1806025421	0.11322051	-1.60	0.1153	-.4064712865	0.0452662022
**Edu2way_stand**	-.1297229693	0.10823869	-1.20	0.2348	-.3456532379	0.0862072993
**Par_stand**	0.0821119293	0.11913729	0.69	0.4930	-.1555604647	0.3197843234
**Mage_stand**	-.0312794786	0.11239797	-0.28	0.7816	-.2555072947	0.1929483375
**GA_stand**	0.1080751948	0.10026163	1.08	0.2848	-.0919412800	0.3080916697
**EPDS_Blm_stand**	-.3305228878	0.11734364	-2.82	0.0063	-.5646170346	-.0964287410
**PPRFQ_Blm_stand**	0.3776791660	0.11740622	3.22	0.0020	0.1434601739	0.6118981581
