# Supplementary material for: Maternal-fetal bonding among pregnant women at psychosocial risk: The roles of adult attachment style, prenatal parental reflective functioning, and depressive symptoms
Source: PLoS One. 2020 Sep 17;15(9):e0239208. doi: 10.1371/journal.pone.0239208 (PMC7498041; doi:10.1371/journal.pone.0239208)
Supplement: Attachment — Submitted filename: PLOS One Reviewer Comments.pdf [file pone.0239208.s003.pdf]

Thank you for the opportunity to review this paper. Rohder and colleagues report findings from their study which examined psychosocial correlates of maternal-fetal bonding amongst at-risk pregnant women. This manuscript contributes new evidence to the field as well as important implications for future perinatal screening and intervention.

I do have some suggestions for the paper.

Abstract

- The study design should be mentioned in the abstract

-Line 24: The sentence starting "The study aim was to study…" Consider the double use of the word 'study'.

-Line 35: Please present the key findings from this study in numerical form.

- Please include the main limitation of the study in the last paragraph of the abstract.

Introduction:

-Overall, the introduction is well-written and comprehensive, however the authors could condense much of this background information, particularly when discussing past research findings and the Maternal Antenatal Attachment Scale. I think the focus could instead be on the parts of the introduction which explain why this study is important, the new information it offers to the field and the justification of the methodological decisions the authors have made.

-Second paragraph of the introduction: the authors talk about emotional well-being and emotional distress during pregnancy but I think it is worthwhile to expand upon this. More specifically, how the perinatal period brings about an increased vulnerability for women for both the onset and recurrence of mental illnesses such as depression. Perinatal depression is highly relevant to this study and to the topic of mother-fetal bonding so it deserves a point of discussion here in the introduction.

Method

Overall this section is clear and well written. I have a few suggestions for improvement below.

- More information on the setting and location of the study would assist readers, especially those unfamiliar with locations in Denmark and the Danish health system. As this was a study recruiting at-risk women, were the locations of these hospitals in areas with high social disadvantage and at-risk populations?

- Line 252: The authors state that risk status was defined by the official Danish Health Care recommendations whereby GPs or midwives identify pregnant women at risk based on known mental health history. How does this information come to be "known"? Is it based on self-report by the woman at time of appointment, health records, or is there some sort of standard mental health screening or psychosocial questionnaire that takes places for women in the health service as part of their pregnancy care?

- Line 256: Considering this study is examining at-risk women, I think it would be beneficial to the reader to offer more details of the criteria for being deemed at-risk. It mentions in the paper severe social vulnerabilities such as limited social network or partner with severe mental illness. What other social vulnerabilities were considered eligible?

- Line 258: I have some concerns with the exclusion criteria. Firstly, it states that those unable to speak or understand Danish were excluded, as well as those who had a previous child placed in care. Both of these factors would be highly prevalent among at-risk women and I am therefore concerned that this study may have excluded a good part of its targeted population and introduced bias which could affect the generalisability of the results. Can the authors offer more of a strong rationale and justification for this perplexing exclusion criteria? This also needs to be discussed in the limitation section of the discussion. It may offer an explanation as to why characteristics of the participants appear to be functioning at a higher level (relationships, educated, and employed) than to be expected for an at-risk population.

- What were the professional backgrounds of the researchers who contacted the women by phone and conducted the home assessments? Was any training undertaken for the researchers in order to administer the measures?

- On average how long did each home interview take?

- Line 275: The authors mention that 61 women chose not to participate for reasons of not needing extra intervention, lack of energy, and not wanting to be video recorded. Can you provide the specific number breakdown for each of these reasons? As a reader, I am particularly interested in how many declined due to not wanting to be video recorded. I am also unsure as to the exact reason why the women participating in the study were to be video recorded, can the authors please provide further explanation.

- It could be worthwhile to use headings for each of the measure administered in this study. It will assist the reader to quickly identify the measures used.

-Line 325: When discussing the details of the EPDS, there is no mention of the items which assess symptoms of anxiety, the resulting anxiety subscale score, as well as question 10 which assesses self-harm. I think this needs to be mentioned and included in the results. If the authors, choose not to do this then a justification needs to be provided as to why this data is not reported.

- Line 326 Please give specific details on the performance (high sensitivity and specificity) for the EPDS.

- Line 329 Should read "cut off point" not cut point

- Line 329 The description of the meaning of the cut off point needs to be clearer. The cut off points are applied to indicate the possibility of risk for probable depression not just the presence of depressive symptoms.

- Was a power analysis conducted prior to the study? Are you able to explain how the study size was arrived at?

- Line 337: Can the authors please explain their choices for controlled variables and expand upon the reasons as to why these variables may confound the results?

Results

- Line 349: Can the authors report the results from the EPDS administration which includes the anxiety subscale. It would be of note to add how many women scored 1 or higher on question 10.

- Table 1: In the heading of table 1 please state the number of participants to show there was no missing data for these questions.

- Table 3: In this table the study results are presented in the format of n (%) but the normative data is not presented in the same format. I am assuming it is percentages presented for the normative data? This needs more clarification, if possible present both the n and % of the normative data.

Discussion

- Line 505: There are some issues with the generalizability of the results and this should be discussed in the limitation section. The authors state that unmeasured differences between women who chose to participate and those who declined participation limit the generalizability of the findings. It should be stated in addition, that the study did have a high non-participation rate. Perhaps as a result of the study's methodology which involved video recording of participants, a somewhat intrusive choice of data collection and not yet justified by the authors in this current manuscript.

-The other limitation which must be addressed is the selection of the study sample which excluded participants based on what many researchers would consider to be key at-risk characteristics, i.e. poor language skills and children in care. The authors need to reflect upon this and offer more of a discussion on how this ultimately affects their results.

Given all of the above, this paper makes some important recommendations regarding the future of prenatal screening and opportunities for intervention to improve parenting practices and ultimately mother-child outcomes. However, this paper needs major revisions but could still be a helpful publication if strengthened.
